# Incidence and risk factors of age-related macular degeneration in patients with Parkinson’s disease: a population-based study

**DOI:** 10.3389/fnagi.2024.1331786

**Published:** 2024-04-19

**Authors:** Bora Yoon, Ho-Seok Sa, Hwa Jung Kim

**Affiliations:** ^1^Department of Neurology, Seoul St. Mary’s Hospital, College of Medicine, The Catholic University of Korea, Seoul, Republic of Korea; ^2^Department of Ophthalmology, ASAN Medical Center, University of Ulsan College of Medicine, Seoul, Republic of Korea; ^3^Department of Clinical Epidemiology and Biostatistics, ASAN Medical Center, University of Ulsan College of Medicine, Seoul, Republic of Korea

**Keywords:** age-related macular degeneration, Parkinson’ disease, incidence, risk factor, epidemiology

## Abstract

**Background and objective:**

The association between age-related macular degeneration (AMD) and Parkinson’ disease (PD) remains unclear. The aim of the present study was to assess the incidence of AMD in patients with PD, elucidate differences by age and sex, and investigate potential risk factors for AMD.

**Methods:**

Data were extracted from the Korean National Health Insurance System database, which covers 97% of the Korean population (2002 through 2019). We calculated the incidence of newly diagnosed AMD in patients with PD and used Cox proportional-hazards models to estimate risk factors for AMD, presenting adjusted hazard ratios (aHR) with 95% confidence intervals (CI).

**Results:**

Of 172,726 patients with PD, 15,800 were newly diagnosed with AMD during the follow-up, including 5,624 men and 10,176 women. The overall incidence of AMD in patients with PD was 13.59 per 1,000 person-years. Stratified by age group and sex, the incidence was higher in women aged 40–69, and conversely higher in men aged 70–89. Risk of AMD was high in older age groups (aHR = 4.36, 95% CI: 3.74–5.09 in the 70 s), female sex (aHR = 1.07, 95% CI: 1.04–1.11), patients with diabetes mellitus (DM) (aHR = 1.14, 95% CI: 1.10–1.18), and patients with hyperlipidemia (aHR = 1.17, 95% CI: 1.13–1.21).

**Conclusion:**

Our findings suggest that the AMD incidence is higher in patients with PD than in the general population, with varying patterns of sex differences across age groups. Particularly, old age, female sex, presence of DM, and hyperlipidemia are potential risk factors. Therefore, clinicians should pay greater attention to AMD in patients with PD.

## Introduction

Parkinson’ disease (PD) is a progressive neurodegenerative disorder primarily characterized by motor dysfunctions such as resting tremor, rigidity, bradykinesia, and gait disturbances (Poewe et al., 2017). As diverse non-motor symptoms, patients with PD present visual impairments (Armstrong, 2017; Ekker et al., 2017) and have a higher prevalence of ocular symptoms compared to controls (Borm et al., 2020). These visual symptoms in PD can range from reduced color discrimination, contrast sensitivity, motion perception, visual processing speeds, visual acuity, and alongside retinal degeneration (Archibald et al., 2009; Bodis-Wollner, 2009; Nowacka et al., 2014; Armstrong, 2017; Ekker et al., 2017). From a retinal perspective, several studies have reported dopaminergic retinal cell loss or retinal alterations in patients with PD (Satue et al., 2017; Ahn et al., 2018; Young et al., 2019; Ortuño-Lizarán et al., 2020; Tugcu et al., 2020). A potential mechanism involves that the loss of dopaminergic cells in the retinal layers in patients with PD, resulting in the accumulation of phosphorylated α-synuclein, leading to progressive retinal degeneration and subsequent visual impairment (Mohana Devi et al., 2020). Furthermore, patients with PD may show reduced macular volume, thinner and broader mean foveal pit, and higher incidence of glaucoma (Bayer et al., 2002; Nowacka et al., 2014, 2017; Ekker et al., 2017). These visual impairments can significantly impact daily activities, resulting in reduced social and physical engagements and an overall lower quality of life (Santos-García and de la Fuente-Fernãndez, 2013).

Age-related macular degeneration (AMD) is a progressive degenerative disease affecting the macula, which is the central region of the retina responsible for sharp, detailed vision. It is characterized by pathological changes in the deeper retinal layers and surrounding vasculature, leading to the loss of central vision (Apte, 2021; Fleckenstein et al., 2021; Thomas et al., 2021). AMD is the leading cause of visual impairment and loss in developed countries among people over 50 years old. It accounts for 6–9% of legal blindness cases globally, placing a significant burden on patients’ quality of life and healthcare costs (Wong et al., 2014). In the prevalence and incidence of AMD, the sex predilection has not been conclusively determined due to conflicting results (Klein et al., 2010; Wong et al., 2014; Park et al., 2015; Maguire et al., 2016; Rim et al., 2019; Thomas et al., 2021). Moreover, variations in the prevalence and incidence of AMD in the general population are observed among different races and ethnicities, with lower rates among Asians than among Caucasians, although variations exist among different Asian subgroups (Kawasaki et al., 2010; Wong et al., 2014). In the context of PD, our current knowledge lacks comprehensive insights into the precise prevalence or incidence of visual impairment. Understanding how factors such as age, sex, race, and demographics contribute to varying rates in the general population highlights a persistent knowledge gap even within the PD patient population. The absence of studies examining prevalence or incidence rates in patients with PD, underscores the need for our research. In addition, various risk factors for AMD have been proposed (Chakravarthy et al., 2010), however, studies have not consistently demonstrated concordant results for modifiable risk factors such as environmental or behavioral factors, in contrast to non-modifiable factors such as age and genetic predisposition (Salimiaghdam et al., 2019). Furthermore, there is currently a lack of research on risk factors for AMD specifically in patients with PD.

Some studies suggest a high risk of PD in patients with AMD (Chung et al., 2014; Etminan et al., 2018; Choi et al., 2020), and indicate that both AMD and PD exhibit thin retinal nerve fibers (Lee and Yu, 2015). In contrast, evidence for the risk of AMD in patients with PD remains limited. Therefore, the aim of the present study was to explore the incidence rates and risk factors for AMD in Koreans with PD and investigated differences by age and sex. We also sought to identify potential risk factors for AMD in patients with PD.

## Materials and methods

### Data source

Data was extracted from the Korean National Health Insurance Service (KNHIS), which covers approximately 97% of the Korean population, from 1 January 2002, to 31 December 2019. Extracted data included personal demographics, socioeconomic status, diagnostic records, procedures, prescription records, and direct medical costs. Diagnoses in KNHIS are registered according to the International Classification of Diseases, 10th Revision (ICD-10) codes (Quan et al., 2005). In South Korea, an enrollment program for rare and intractable diseases (RIDs) has been implemented to alleviate the economic burden of patients with rare diseases. PD is registered as a RID, with a unique diagnostic code (code V) assigned in conjunction with the ICD-10 code (V124). The V code is assigned following stringent diagnostic criteria set by a certified neurologist, ensuring high reliability. The study protocol was approved by the Institutional Review Board (IRB) of Konyang University Hospital (no. KYUH 2021-05-026). As the KNHIS dataset is subject to stringent confidentiality regulations set by the Korean government, and data provided to researchers have been anonymized, the requirement for obtaining written informed consent was waived by the IRB.

### Study population

**FIGURE 1:**
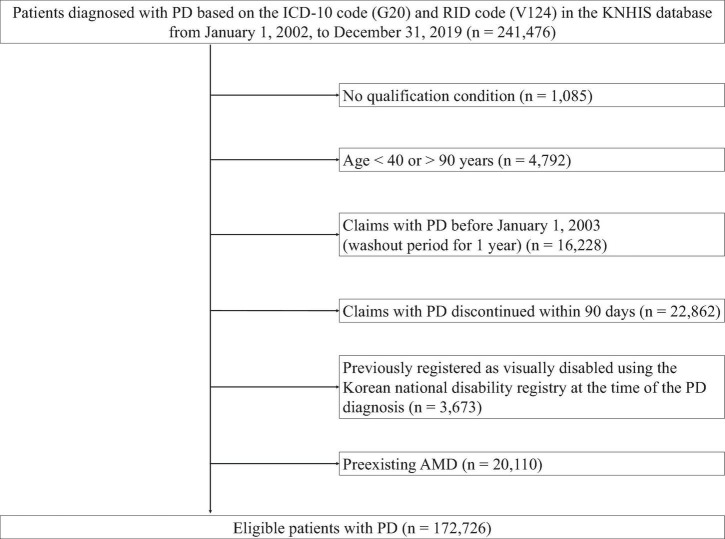
Flow chart of the study population. PD, Parkinson’ disease; ICD-10, International Classification of Diseases, 10th revision; RID, rare and intractable disease; KNHIS, Korean National Health Insurance Service.

A retrospective cohort of patients diagnosed with PD in the KNHIS database, was established. The diagnosis was based on the ICD-10 (G20) and RID (V124) codes for PD. The index date was the date of the initial PD diagnosis. A total of 241,476 patients with PD were identified. Exclusion criteria were: lack of qualifications, indicating missing information on important demographic factors such sex, age, socioeconomic status (including residence and income) (*n* = 1,085); aged < 40 or > 90 years (*n* = 4,792), first PD claim before 1 January 2003, as a wash-out period (*n* = 16,228); discontinued PD claims within 90 days from the index date (*n* = 22,862); registration as a visually disabled patient using the Korean National Disability Registry before the PD diagnosis (*n* = 3,673); and pre-existing AMD (*n* = 20,110). AMD diagnosis was established by an ophthalmologist. Visually disabled patients registered in the Korean National Disability Registry are defined as individuals who meet stringent criteria, including comprehensive medical records, objective examinations by specialized ophthalmologists, and confirmation through diagnosis certificates, even after 6 months of treatment, to ensure permanence of the impairment. Patients with pre-existing AMD, based on the ICD-10 code (H35.3), at the time of the initial PD diagnosis were excluded considering the primary objective of estimating the incidence of newly developed AMD. After applying these exclusion criteria, 172,726 patients with PD were included in this study. Figure 1 illustrates the flowchart of patient selection.

### Clinical variables

Age-related macular degeneration (AMD) was defined as the ICD-10 code H35.3 (Quan et al., 2005). Information on pre-existing comorbidities within the past year from the initial claim dates of PD was also collected. Modifiable risk factors encompassing diet, physical activity, smoking, and metabolic risk factors such as hypertension, atherosclerosis, stroke, myocardial infarction, atrial fibrillation (AF), diabetes mellitus (DM), high body mass index (BMI), higher plasma fibrinogen, and hyperlipidemia are considered. Although a number of studies have shown a link between cardiovascular risk factors and AMD, the roles of hypertension, atherosclerosis, high BMI, DM, hyperlipidemia, and so on remain inconclusive due to inconsistent findings (Fleckenstein et al., 2021). In this study, we included hypertension (I10-13, and I15), DM (E10-14), hyperlipidemia (E78), AF (I48), and stroke (I60-64, and G45) as potential confounders, taking into account feasibility and minimizing susceptibility to bias.

### Statistical analyses

Categorical variables are expressed as count/frequency (%), and were compared using the chi-square test. Additionally, standardized differences were calculated to determine the balance of baseline covariates between the two groups (Austin, 2009). The incidence rate of AMD was calculated as the number of cases per person-years (PY) at risk. The person-time incidence rates from 2002 to 2019 were calculated as the number of people who developed AMD, divided by the total person-time at risk during the study period. The observation period in years is presented as both median (first quartile [Q1] and third quartile [Q3]) and mean (standard deviation). To compare the incidence rate to the nonPD patients, we adopted indirect standardization using data of Rim et al. (2019) as the reference population of age-specific rates to calculate the expected numbers of AMD cases among PD population.

The Kaplan–Meier (KM) curves were employed to graphically represent the survival function. The Cox proportional-hazards regression analysis was used to determine the hazard ratio (HR) for AMD, *p*-value, and 95% confidence interval (CI). The Schoenfeld plots were used to assess the proportionality assumption (data not presented). Three adjusted models were fitted for the analyses. Model 1 included adjustments for age and sex as a categorical variable. Model 2 was adjusted for age, sex, and all comorbidities. The interaction between age and sex was examined, and upon confirmation, we implemented sex-stratified models. We conducted both univariate Cox proportional-hazards regression analysis (model 3) and multivariate analysis adjusting for age and all comorbidities (model 4). As we included 172,726 patients with PD and AMD was observed among approximately 10% of them, we believed that the power would be sufficient for conducting statistical analysis, considering the application of the “event-per-variable” (EPV) ratio (Heinze et al., 2018). Therefore, we did not need to perform statistical selection using stepwise backward or forward selection algorithms. All candidate risk factors selected clinically were included in the full model. Statistical analyses were conducted using SAS version 9.4 (SAS Institute, Cary, NC, USA) and R software version 4.0.5 (R Foundation for Statistical Computing, Vienna, Austria).^1^ A *p*-value ≤ 0.05 was deemed statistically significant for all two-sided tests.

## Results

### Population characteristics

Table 1 presents the baseline characteristics of the study population. In the final analysis, we enrolled 172,726 patients with PD, including 72,793 (42.1%) men and 99,933 (57.9%) women. Throughout the total follow-up period, 15,800 of 172,726 (9.2%) patients were newly diagnosed with AMD. The frequency of PD diagnoses was the highest among patients in their 70 s, followed by those in their 60 s, regardless of sex. Similarly, the frequency of newly diagnosed AMD was the highest among patients in their 70 s, followed by those in their 60 s (Table 1). Compared to PD patients without AMD at the end of the study period, those with AMD were more likely to be female sex, older, or have more comorbidities, such as hypertension, DM, hyperlipidemia, AF, and stroke (all *p* < 0.05) (Table 1). Specifically, the prevalence of hypertension, DM, and hyperlipidemia was significantly higher among PD patients with AMD than among those without AMD, regardless of sex (Table 1). In the prevalence of stroke and AF, an observed difference in *p*-values noted among male patients with PD and female patient with PD groups based on the presence of AMD. However, since the SMD was less than 0.1 and KM curves also demonstrated no significant differences between the groups, the observed differences in *p*-value of stroke and AF were not considered statistically significant (Table 1).

**TABLE 1:** Demographic characteristics of 172,726 patients with PD.

	Non-AMD	AMD	*p*	SMD	Males (*n* = 72,793)				Females (*n* = 99,933)			
					Non-AMD	AMD	*p*	SMD	Non-AMD	AMD	*p*	SMD
Total number	156,926	15,800			67,169	5,624	< 0.001	0.250	89,757	10,176	< 0.001	0.309
Women, *n* (%)	89,757 (57.2)	10,176 (64.4)	< 0.001	0.148								
Age group at the PD diagnosis			< 0.001	0.281								
40–49 years	5,061 (3.2)	167 (1.1)			2,771 (4.1)	85 (1.5)			2,290 (2.6)	82 (0.8)		
50–59 years	16,759 (10.7)	1,175 (7.4)			8,324 (12.4)	458 (8.1)			8,435 (9.4)	717 (7.0)		
60–69 years	41,635 (26.5)	5,236 (33.1)			18,847 (28.1)	1,817 (32.3)			22,788 (25.4)	3,419 (33.6)		
70–79 years	67,948 (43.3)	7,563 (47.9)			27,627 (41.1)	2,627 (46.7)			40,321 (44.9)	4,936 (48.5)		
80–89 years	25,523 (16.3)	1,659 (10.5)			9,600 (14.3)	637 (11.3)			15,923 (17.7)	1,022 (10.0)		
**Comorbidities**												
Hypertension	99,755 (63.6)	10,493 (66.4)	< 0.001	0.060	40,441 (60.2)	3,587 (63.8)	< 0.001	0.074	59,314 (66.1)	6,906 (67.9)	< 0.001	0.038
DM	60,328 (38.4)	6,644 (42.1)	< 0.001	0.074	25,522 (38.0)	2,376 (42.3)	< 0.001	0.087	34,806 (38.8)	4,268 (41.9)	< 0.001	0.065
Hyperlipidemia	75,152 (47.9)	8,123 (51.4)	< 0.001	0.070	30,123 (44.8)	2,680 (47.7)	< 0.001	0.056	45,029 (50.2)	5,443 (53.5)	< 0.001	0.066
AF	6,033 (3.8)	534 (3.4)	0.004	0.025	2,948 (4.4)	247 (4.4)	> 0.999	< 0.001	3,085 (3.4)	287 (2.8)	0.001	0.035
Stroke	49,748 (31.7)	4,798 (30.4)	0.001	0.029	22,385 (33.3)	1,776 (31.6)	0.008	0.037	27,363 (30.5)	3,022 (29.7)	0.104	0.017

AMD, age-related macular degeneration; SMD, standardized mean difference; PD, Parkinson’ disease; DM, diabetes mellitus; AF, atrial fibrillation. Data are expressed as number (%). *P*-values were obtained by chi-square test for categorical variables.

### Incidence and risk factors of AMD in patients with PD

During the study period, the overall incidence rate of AMD among patients with PD was 13.59/1,000 PY (Table 2). When analyzed by age group, the incidence rates of AMD increased almost twofold every decade, from 3.26/1,000 PY in the 40 s, to 7.64/1,000 PY in the 50 s, and 14.56/1,000 PY in the 60 s. From the 60 to 80 s, the incidence rates of AMD remained relatively constant (Table 2). When stratified by age group and sex, the incidence rate of AMD was the highest among women aged 40–69 years old, whereas it was the highest among men aged 70–89 years old (Table 2). Women with PD had a higher incidence of AMD compared to men in their 50 s (8.78 vs. 6.35/1,000 PY) and 60 s (15.78 vs. 12.70/1,000 PY; Table 2). Among PD patients with AMD, the age at PD diagnosis was inversely proportional to the median time until the occurrence of AMD. Specifically, patients diagnosed with PD in their 40, 50, 60, 70, and 80 s took approximately 7 (median [Q1–Q3]: 7.17 [3.13–10.64]), 6 (6.12 [2.88–9.62]), 4.5 (4.49 [2.07–7.73]), 3 (2.86 [1.28–5.43]), and 2 (1.85 [0.82–3.63]) years to develop AMD, respectively. When stratified by sex, men diagnosed with PD in their 40 s (5.38 [2.23–9.96]) and 50 s (5.42 [2.30–9.96]) took approximately 5 years to develop AMD, while women diagnosed with PD in their 40 and 50 s took approximately 8 (8.23 [3.5–11.28]) and 7 (6.58 [3.13–9.69]) years, respectively. The median time until the development of AMD was longer in women than in men in the middle-aged group, but did not differ significantly between the sexes, from the age of 60 years onward. After conducting indirect comparisons using the standardized incidence rate (SIR) to compare incidence from the general population data, considering age groups (by 5 years intervals) and sex as reported by Rim et al. (2019), Koreans with PD showed a significantly higher incidence of AMD compared to the general population (the SIR as 19.79 [19.48–20.10], *p* < 0.001).

**TABLE 2:** Incidence of AMD among patients with PD.

	Total				Male				Female			
Age group	Cases	Observation period (year)		Incidence	Cases	Observation period (year)		Incidence	Cases	Observation period (year)		Incidence
		Median [Q1-Q3]	Mean (SD)			Median [Q1-Q3]	Mean (SD)			Median [Q1-Q3]	Mean (SD)	
Total	15,800	5.86 [3.43–9.40]	6.73 (4.25)	13.59	5,624	5.22 [3.08–8.43]	6.12 (4.02)	12.63	10,176	6.38 [3.75–10.07]	7.17 (4.36)	14.19
40–49	167	9.49 [5.68–13.83]	9.81 (4.82)	3.26	85	9.03 [5.33–13.49]	9.45 (4.80)	3.15	82	10.08 [6.15–14.29]	10.24 (4.80)	3.37
50–59	1,175	7.91 [4.80–12.02]	8.57 (4.58)	7.64	458	7.54 [4.55–11.43]	8.21 (4.47)	6.35	717	8.30 [5.09–12.54]	8.92 (4.65)	8.78
60–69	5,236	6.87 [4.07–10.88]	7.67 (4.47)	14.56	1,817	6.10 [3.70–9.64]	6.93 (4.15)	12.70	3,419	7.60 [4.45–11.82]	8.27 (4.62)	15.78
70–79	7,563	5.54 [3.25–8.66]	6.24 (3.89)	16.05	2,627	4.72 [2.83–7.39]	5.38 (3.43)	16.14	4,936	6.18 [3.63–9.50]	6.82 (4.07)	16.00
80–89	1,659	4.05 [2.35–6.32]	4.65 (3.10)	13.14	637	3.45 [1.91–5.35]	3.95 (2.71)	15.77	1,022	4.50 [2.68–6.92]	5.07 (3.25)	11.90

Q1, the first quartile; Q3, the third quartile; SD, standard deviation. The values of incidence represent per 1,000 person-years (PY). The observation period (year) was presented as median [Q1-Q3] and mean (SD).

**FIGURE 2:**
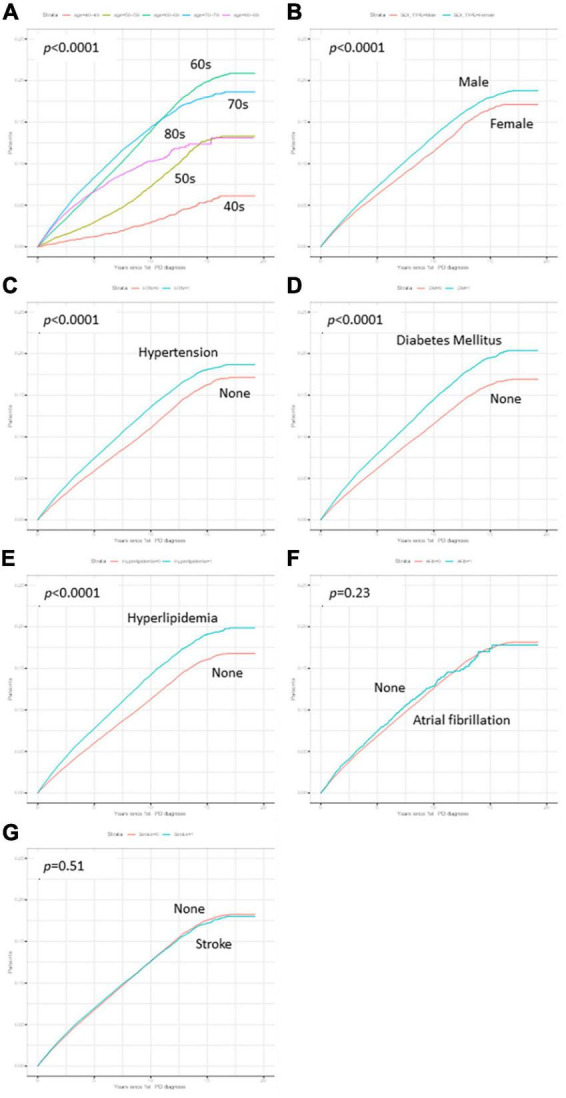
Kaplan–Meier curves of variables. The cumulative survival probabilities were different according to time by age decades **(A)**, sex **(B)**, hypertension **(C)**, Diabetes mellitus (DM) **(D)**, and hyperlipidemia **(E)**, while the cumulative survival probabilities showed no differences in atrial fibrillation **(F)** and stroke **(G)**.

**FIGURE 3:**
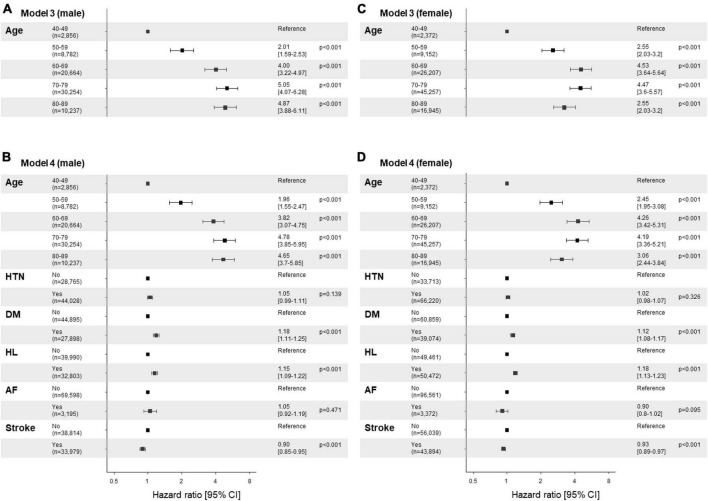
Risk factor plots stratified by sex. **(A-D)** Forest plots display the results of the multivariable analysis using the Cox-proportional hazards model stratified by sex (**A,B**; male vs. **C,D**; female). The figures utilize a logarithmic scale, and the horizontal bars on the plots represent the 95% confidence intervals (CI) for each variable. Panels **(A,C)** display the results of univariate analysis by age, while panels **(B,D)** show the results of the multivariable analysis adjusted by age and comorbidities, including hypertension, diabetes mellitus, hyperlipidemia, atrial fibrillation, and stroke. The corresponding *p*-values are provided for each variable, along with the precise adjusted hazard ratio (aHR) and the 95% CIs enclosed in square brackets. HTN, hypertension; DM, diabetes mellitus; HL, hyperlipidemia; AF, atrial fibrillation.

The cumulative survival probabilities varied with time across age decades, sex, HTN, DM, and hyperlipidemia (Figure 2). Patients with PD had a high risk of AMD in both models 1 and 2 (Table 3), considering the adjusted HR (aHR) and 95% CI. The risk of AMD was high among female sex (model 2: 1.07 [1.04–1.11]), and patients with DM (model 2: 1.14 [1.10–1.18]), and hyperlipidemia (model 2: 1.17 [1.13–1.21]) (Figure 3B). Additionally, in model 2, the aHR was the highest in the patients in their 70 s (4.36 [3.74–5.09]), followed by those in their 60 s (4.08 [3.50–4.76]) in reference to those in their 40 s (Table 3). Likewise, the aHR for AMD did not differ significantly between models 1 and 2 (females: 1.09 [1.05–1.12], patients in 70 s: 4.63 [3.97–5.40], patients in 60 s: 4.31 [3.69–5.02]) (Table 3). In the sex-stratified analysis of model 4 (Figure 3), examining the risk factors for AMD, we observed similar trends to those seen in model 2. Irrespective of sex, as age increased, patients with DM and hyperlipidemia consistently exhibited a higher aHR (Figures 3B, D). Specifically, among females, the aHRs in their 60 s (4.26 [3.42–5.31]) exceeded those in their 70 s (4.19 [3.36–5.21]) and 80 s (3.06 [2.44–3.84]) (Figure 3D), while among males, the aHR in their 70 s (4.78 [3.85–5.95]) was highest compared to those in their 60 s (3.82 [3.07–4.75]) and 80 s (4.65 [3.70–5.85]), with reference to the 40 s age group (Figure 3B). In the 50 s age group, women exhibited a relatively higher aHR (2.45 [1.95–3.08]) than those of men in their 50 s (1.96 [1.55–2.47]) (Figures 3B, D). Additionally, we conducted Cox proportional-hazard analysis with age as a continuous variable. The results revealed a 3% rise in the incidence of AMD among males and a corresponding 1% increase among females.

**TABLE 3:** Multivariable Cox proportional-hazard model for AMD among patients with PD.

		(A) Model 1			(B) Model 2		
**Variable**		**HR**	**95% CI**	***p***	**HR**	**95% CI**	***p***
Sex	Male	Reference	1.05–1.12	Reference	1.07	1.04–1.11	< 0.001
	Female	1.09		< 0.001			
Age	40–49	Reference		Reference			
	50–59	2.30	1.96–2.70	< 0.001	2.23	1.90–2.62	< 0.001
	60–69	4.31	3.69–5.02	< 0.001	4.08	3.50–4.76	< 0.001
	70–79	4.63	3.97–5.40	< 0.001	4.36	3.74–5.09	< 0.001
	80–89	3.70	3.15–4.34	< 0.001	3.52	3.00–4.13	< 0.001
HTN					1.03	0.99–1.06	0.158
DM					1.14	1.10–1.18	< 0.001
HL					1.17	1.13–1.21	< 0.001
AF					0.97	0.89–1.06	0.492
Stroke					0.91	0.87–0.94	< 0.001

HR, hazard ratio; CI, confidence intervals; HTN, hypertension; DM, diabetes mellitus; HL, hyperlipidemia; AF, atrial fibrillation. Model 1 was adjusted for age and sex, and model 2 was adjusted for age, sex, and comorbidities including HTN, DM, HL, AF, and stroke. *P*-values were derived from the Cox proportional-hazard model.

## Discussion

We confirmed the incidence rate and risk factors for AMD in Koreans with PD, considering variations by age group and sex. The incidence rate of AMD is naturally expected to increase with age in patients with PD. Factors such as race and ethnicity, as reported in meta-analyses (Clemons et al., 2005; Klein et al., 2007; Etminan et al., 2018), showed varying prevalence and incidence rates, with higher rates among Caucasians than among Asians. A meta-analysis estimated the annual incidence of late AMD in white American individuals aged over 50 years to be 3.5/1,000 PY (95% CI; 2.5–4.7/1,000 PY) (Rudnicka et al., 2015). Previous studies using the KNHIS database showed that the 3-year incidence of AMD from 2010 to 2012 was 0.30 (95% CI; 0.30–0.31)/1,000 PY (Park et al., 2015) and the 6-year incidence from 2010 to 2015 was 0.29 (95% CI; 0.28–0.30)/1,000 PY (Rim et al., 2019) in the general Korean population. However, the overall incidence rate (13.59/1,000 PY) which were a significantly higher incidence rate of AMD in patients with PD (i.e., as the SIR were observed to be 19.79 [19.48–20.10]), compared to the general Korean population aged ≥ 40 years. This outcome is consistent with a study using Medicare claims to evaluate the prevalence of visual impairment in the PD and non-PD populations (Hamedani et al., 2020). The study revealed that PD was independently linked to a higher risk of moderate-to-severe visual impairment (1.67% in PD vs. 0.71% in non-PD), even following adjustments for variables such as age, race, sex, and socioeconomic status. Furthermore, the study identified prevalent ocular comorbidities in PD: cataracts (36.3%), AMD (18.9%), and glaucoma (14.9%), mirroring their prevalence in the general population (Hamedani et al., 2020).

As the age at which AMD is diagnosed can vary, the incidence rate was observed to follow a reverse-J shape in both PD and non-PD populations. The incidence rate was lowest among individuals in their 40 s and increased as they got older until their 70 s. It then slightly decreased in their 80 s. This disparity was particularly pronounced among patients in their 40 and 50 s, which represent a relatively younger age group, and gradually decreased with increasing age regardless of sex. Specifically, the study conducted by Rim et al. (2019) reported minimal incidences for the 40–44 age group (0.01, 95% CI; 0.00–0.02) and for the 45–49 age group (0.02, 95% CI; 0.01–0.04)/1,000 PY. Similarly, the incidence for the age group 50–54 years and 55–59 years were reported at 0.10 (95% CI 0.08–0.13) and 0.15 (95% CI 0.12–0.19)/1,000 PY, respectively (Rim et al., 2019). In contrast, our results revealed higher incidences of 3.26/1,000 PY in the 40–49 age group and 7.64/1,000 PY in the 50–59 age group of patients with PD, highlighted an extremely low incidence rate of AMD in these age groups within the general population.

Among Koreans (Park et al., 2015; Rim et al., 2019), the incidence of AMD was higher in men than in women across all age groups, with a male-to-female ratio of 1.6. Wong et al. (2014) reported that women are more susceptible to developing AMD compared to men with female preponderance (Maguire et al., 2016), but some studies reported the opposite results (Klein et al., 2010; Thomas et al., 2021), making the sex predilection inconclusive. In the present study, patients with PD showed a trend of the highest incidence rates of AMD among women aged 40–69 years and men aged 70–89 years. In the general population, factors pertaining to female reproductive history, such as an extended reproductive period and prolonged exposure to estrogen, have been linked to a heightened risk of AMD (Hwang et al., 2021). Additionally, in postmenopausal women, exposure to both endogenous and exogenous female hormones could influence the risk of AMD prevalence and progression (Baek and Lee, 2017; Yuk and Hwang, 2022). Considering these potential connections, the higher risk of AMD in relatively young women patients with PD could be attributed to the association with female hormones.

The Cox-proportional hazards models revealed that patients with PD had a higher risk for AMD among female sex, patients with DM, and those with hyperlipidemia. Although age is a widely acknowledged primary risk factor for AMD, it is important to note that younger female patients with PD, compared to males, specifically those below 70 years old, demonstrated an increased risk for AMD. The roles of hypertension, atherosclerosis, high body mass index, DM, and hyperlipidemia remain equivocal owing to inconsistent findings (Clemons et al., 2005; Rudnicka et al., 2015; Mitchell et al., 2018; Heesterbeek et al., 2020; Fleckenstein et al., 2021). In the Cox-proportional hazard model, neither hypertension, stroke nor AF were confirmed as a risk factor for AMD in patients with PD. This aligns with conflicting findings regarding the association between AMD and cardiovascular disease or stroke (Pennington and DeAngelis, 2016).

Our results suggest that DM and hyperlipidemia increase the risk of AMD in patients with PD. The study demonstrating the association between hyperlipidemia and AMD as a risk factor is in accordance with our results (Pennington and DeAngelis, 2016). On the other hand, the relationship between DM and diabetic retinopathy (DR) with AMD is contentious, with some suggesting an increased prevalence of AMD (Yongpeng et al., 2022; Hwang et al., 2023), other finding no association (Voutilainen-Kaunisto et al., 2000), and some even proposing a potential protective effect of DM or DR against AMD (Srinivasan et al., 2017). Our findings, indicating DM as a risk factor for AMD, are in line with the plausible consideration that chronic inflammatory process driven by DM contribute to both DR and AMD by compromising the retinal/choroidal vasculature and causing blood-retinal barrier breakdown, potentially leading to hypoxia in the outer retina and an increased risk of AMD (Adamiec-Mroczek et al., 2010; Klaassen et al., 2013).

The precise mechanisms underlying the association between PD and AMD remain unclear, but pathogenetic factors that could elucidate the link between them are shared. Chronic inflammation and subsequent neurodegeneration are associated with pathogeneses of AMD and PD (Buschini et al., 2011; Pajares et al., 2020). The autophagy pathway has been dysregulated in both diseases, leading to the accumulation of misfolded proteins, such as α-synuclein and PINK1-PARKIN in the neurons of patients with PD and lipofuscin in retinal pigment epithelium (RPE) cells of patients with AMD (Delori et al., 2001; Lynch-Day et al., 2012). These pathologic processes could increase oxidative stress and eventually cause structural damage of neural tissues of the retina and brain. Retinal changes in PD, such as thinning of the retina, may contribute to the development of AMD by altering the structure and function of the retina (Archibald et al., 2011; Moschos et al., 2011; Satue et al., 2014).

In particular, retinal thinning can lead to the loss of RPE cells, which are important for maintaining the retinal health, and RPE dysfunction may cause the formation of drusen, which are small yellowish deposits in the retina and a hallmark feature of AMD (Fleckenstein et al., 2021; Flores et al., 2021). Satue et al. (2014) observed that patients with both PD and AMD had greater retinal thinning than those with PD alone. Another possibility is that the deposition of α-synuclein in the retina may impair the function of RPE cells, resulting in the accumulation of lipofuscin and drusen. In patients with PD, α-synuclein accumulates in the outer nuclear layer and photoreceptor cells of the retina, leading to inflammation, oxidative stress, and neuronal death, ultimately increasing the risk of AMD (Ortuño-Lizarán et al., 2018).

In addition to these possibilities, both PD and AMD are multifactorial diseases and share common underlying mechanisms that could contribute to the association. The development of AMD is attributed to various factors, including genetic susceptibility, aging-related dysregulation of normal retinal homeostasis, impaired lipid metabolism, abnormal immune activation, chronic inflammation accompanied by oxidative stress, and dysfunction of the extracellular matrix (Fleckenstein et al., 2021). Chronic inflammation and oxidative stress are involved in the pathogenesis of AMD, potentially contributing to the breakdown of the blood-retina barrier, infiltration of immune cells, and degeneration of retinal neurons in the eye (Mitchell et al., 2018; Fleckenstein et al., 2021). Chronic inflammation and oxidative stress are also involved in the pathogenesis of PD (Miller et al., 2009).

This study has several limitations. First, we did not assess potential risk factors for AMD, such as environmental, lifestyle-related, and dietary factors, including BMI, smoking status, alcohol consumption, physical activities, or dietary habits because of the infeasibility of accurate information or datasets that did not belong to the same cohort. Second, the diagnoses of PD and most comorbidities were determined based on ICD-10 codes, referred to a 2017 report from the Korea Disease Control and Prevention Agency, in which the sensitivity of ICD code ranged from 76.9 to 88.5% compared to the medical chart. Therefore, we had no information on the severity, type, or progression of PD, or how the comorbidities were managed. Third, the use of claims data might have resulted in an underestimation of the incidence of the disease or potential susceptibility to errors. Lastly, as this study used Korean claims data, the findings’ generalizability to other geographic areas, races, or ethnicities may be limited. However, the strength of the present study lies in its utilization of data encompassing all Koreans with PD for a relatively long period from 2002 to 2019. This includes considerations of age- and sex-stratified incidences, the age at the initial PD diagnosis, and associated risk factors.

In conclusion, PD is associated with an increased risk of AMD in the Korean population, regardless of age and sex. Patients with early-onset PD, particularly those in their 40 or 50 s, are at a significantly high risk of developing AMD. This can result in vision impairment within 10 years of the PD diagnosis, particularly earlier in women. Additionally, the female sex and the presence of DM or hyperlipidemia could serve as potential risk factors for AMD in patients with PD. Considering that PD could be a risk factor for AMD, special attention should be paid to young patients with PD. Regular eye check-ups should be considered even in the absence of definite visual symptoms or signs.

## Data availability statement

The Korea National Health Insurance (KNHI) Sharing Service Institutional Data Access/Ethics Committee provides access to confidential data for eligible researchers (https://nhiss.nhis.or.kr/bd/ay/bdaya001iv.do). To apply for this service, researchers should first receive IRB approval from their institution. Upon approval, they may request data access, and following assessment by the KNHI Sharing Service Institutional Data Access/Ethics Committee, are obliged to pay for the cost of data access and usage. Other researchers seeking access to the data should apply for data use in similar manner.

## Ethics statement

The studies involving humans were approved by the Institutional Review Board (IRB) of Konyang University Hospital. The studies were conducted in accordance with the local legislation and institutional requirements. Written informed consent for participation from the participants or the participants’ legal guardians/next of kin was not required in accordance with the local legislation and institutional requirements.

## Author contributions

BY: Conceptualization, Project administration, Writing – original draft. H-SS: Conceptualization, Writing – original draft. HJK: Conceptualization, Data curation, Formal analysis, Methodology, Supervision, Visualization, Writing – original draft.

## Footnotes

1
www.r-project.org

## References

[B1] Adamiec-Mroczek J. Oficjalska-Młyńczak J. Misiuk-Hojło M. (2010). Roles of endothelin-1 and selected proinflammatory cytokines in the pathogenesis of proliferative diabetic retinopathy: Analysis of vitreous samples. *Cytokine* 49 269–274. 10.1016/j.cyto.2009.11.004 20015663

[B2] Ahn J. Lee J. Y. Kim T. W. Yoon E. J. Oh S. Kim Y. K. (2018). Retinal thinning associates with nigral dopaminergic loss in de novo Parkinson disease. *Neurology* 91 e1003–e1012. 10.1212/WNL.0000000000006157 30111550

[B3] Apte R. S. (2021). Age-related macular degeneration. *N. Engl. J. Med.* 385 539–547. 34347954 10.1056/NEJMcp2102061 PMC9369215

[B4] Archibald N. K. Clarke M. P. Mosimann U. P. Burn D. J. (2009). The retina in Parkinson’s disease. *Brain* 132 1128–1145. 19336464 10.1093/brain/awp068

[B5] Archibald N. K. Clarke M. P. Mosimann U. P. Burn D. J. (2011). Retinal thickness in Parkinson’s disease. *Parkinsonism Relat. Disord.* 17 431–436. 21454118 10.1016/j.parkreldis.2011.03.004

[B6] Armstrong R. A. (2017). Visual dysfunction in Parkinson’s Disease. *Int. Rev. Neurobiol.* 134 921–946. 28805589 10.1016/bs.irn.2017.04.007

[B7] Austin P. C. (2009). Using the standardized difference to compare the prevalence of a binary variable between two groups in observational research. *Commun. Stat. Simulat. Comput.* 38 1228–1234.

[B8] Baek S. K. Lee Y. H. (2017). Female hormone factors associated with age-related macular degeneration in menopausal korean women: Knhanes V. *J. Korean Ophthalmol. Soc.* 58 1066–1073.

[B9] Bayer A. U. Keller O. N. Ferrari F. Maag K. P. (2002). Association of glaucoma with neurodegenerative diseases with apoptotic cell death: Alzheimer’s disease and Parkinson’s disease. *Am. J. Ophthalmol.* 133 135–137. 11755850 10.1016/s0002-9394(01)01196-5

[B10] Bodis-Wollner I. (2009). Retinopathy in Parkinson Disease. *J. Neural Transm.* 116 1493–1501. 19730784 10.1007/s00702-009-0292-z

[B11] Borm C. D. J. M. Visser F. Werkmann M. De Graaf D. Putz D. Seppi K. (2020). Seeing ophthalmologic problems in Parkinson disease. *Neurology* 94:e1539. 10.1212/WNL.0000000000009214 PMC7251522 32161030

[B12] Buschini E. Piras A. Nuzzi R. Vercelli A. (2011). Age related macular degeneration and drusen: Neuroinflammation in the retina. *Prog. Neurobiol.* 95 14–25. 21740956 10.1016/j.pneurobio.2011.05.011

[B13] Chakravarthy U. Wong T. Y. Fletcher A. Piault E. Evans C. Zlateva G. (2010). Clinical risk factors for age-related macular degeneration: A systematic review and meta-analysis. *Bmc Ophthalmol.* 10:31. 10.1186/1471-2415-10-31 21144031 PMC3009619

[B14] Choi S. Jahng W. J. Park S. M. Jee D. (2020). Association of age-related macular degeneration on Alzheimer or Parkinson Disease: A retrospective cohort study. *Am. J. Ophthalmol.* 210 41–47. 31712068 10.1016/j.ajo.2019.11.001

[B15] Chung S. D. Ho J. D. Hu C. C. Lin H. C. Sheu J. J. (2014). Increased risk of Parkinson disease following a diagnosis of neovascular age-related macular degeneration: A retrospective cohort study. *Am. J. Ophthalmol*, 157 464–469.e1. 10.1016/j.ajo.2013.09.026 24315292

[B16] Clemons T. E. Milton R. C. Klein R. Seddon J. M. Ferris F. L. III (2005). Risk factors for the incidence of advanced age-related macular degeneration in the age-related eye disease study (Areds) Areds report no. 19. *Ophthalmology* 112 533–539. 10.1016/j.ophtha.2004.10.047 15808240 PMC1513667

[B17] Delori F. C. Goger D. G. Dorey C. K. (2001). Age-related accumulation and spatial distribution of lipofuscin in RPE of normal subjects. *Invest. Ophthalmol. Vis. Sci.* 42 1855–1866. 11431454

[B18] Ekker M. S. Janssen S. Seppi K. Poewe W. De Vries N. M. Theelen T. (2017). Ocular and visual disorders in Parkinson’s disease: Common but frequently overlooked. *Parkinsonism Relat. Disord.* 40 1–10. 28284903 10.1016/j.parkreldis.2017.02.014

[B19] Etminan M. Samii A. He B. (2018). Risk of Parkinson’s disease in patients with neovascular age-related macular degeneration. *J. Curr. Ophthalmol.* 30 365–367. 30555972 10.1016/j.joco.2018.08.004 PMC6277243

[B20] Fleckenstein M. Keenan T. D. L. Guymer R. H. Chakravarthy U. Schmitz-Valckenberg S. Klaver C. C. (2021). Age-related macular degeneration. *Nat. Rev. Dis. Primers* 7:31. 10.1038/s41572-021-00265-2 PMC12878645 33958600

[B21] Flores R. Carneiro Â Vieira M. Tenreiro S. Seabra M. C. (2021). Age-related macular degeneration: Pathophysiology, management, and future perspectives. *Ophthalmologica* 244 495–511. 34130290 10.1159/000517520

[B22] Hamedani A. G. Abraham D. S. Maguire M. G. Willis A. W. (2020). Visual impairment is more common in Parkinson’s disease and is a risk factor for poor health outcomes. *Mov. Disord.* 35 1542–1549. 10.1002/mds.28182 32662528 PMC8183672

[B23] Heesterbeek T. J. Lorës-Motta L. Hoyng C. B. Lechanteur Y. T. E. Den Hollander A. I. (2020). Risk factors for progression of age-related macular degeneration. *Ophthalmic Physiol. Opt.* 40 140–170. 32100327 10.1111/opo.12675 PMC7155063

[B24] Heinze G. Wallisch C. Dunkler D. (2018). Variable selection – A review and recommendations for the practicing statistician. *Biometrical J.* 60 431–449. 10.1002/bimj.201700067 PMC5969114 29292533

[B25] Hwang S. Kang S. W. Han J. Han K. Kim D. Lee K. N. (2021). Female reproductive factors and the risk of exudative age-related macular degeneration: A Nationwide Cohort Study. *Retina* 41 2088–2097. 10.1097/IAE.0000000000003164 33675332

[B26] Hwang S. Kang S. W. Kim S. J. Lee K. N. Han K. Lim D. H. (2023). Diabetes-related risk factors for exudative age-related macular degeneration: A nationwide cohort study of a diabetic population. *Invest. Ophthalmol. Vis. Sci.* 64:10. 10.1167/iovs.64.10.10 37432847 PMC10351020

[B27] Kawasaki R. Yasuda M. Song S. J. Chen S. J. Jonas J. B. Wang J. J. (2010). The prevalence of age-related macular degeneration in Asians: A systematic review and meta-analysis. *Ophthalmology* 117 921–927. 20110127 10.1016/j.ophtha.2009.10.007

[B28] Klaassen I. Van Noorden C. J. Schlingemann R. O. (2013). Molecular basis of the inner blood-retinal barrier and its breakdown in diabetic macular edema and other pathological conditions. *Prog. Retin. Eye Res.* 34 19–48. 10.1016/j.preteyeres.2013.02.001 23416119

[B29] Klein R. Cruickshanks K. J. Nash S. D. Krantz E. M. Nieto F. J. Huang G. H. (2010). The prevalence of age-related macular degeneration and associated risk factors. *Arch. Ophthalmol.* 128 750–758. 20547953 10.1001/archophthalmol.2010.92 PMC2896217

[B30] Klein R. Klein B. E. K. Knudtson M. D. Meuer S. M. Swift M. Gangnon R. E. (2007). Fifteen-year cumulative incidence of age-related macular degeneration: The beaver dam eye study. *Ophthalmology* 114 253–262. 10.1016/j.ophtha.2006.10.040 17270675

[B31] Lee E. K. Yu H. G. (2015). Ganglion cell-inner plexiform layer and peripapillary retinal nerve fiber layer thicknesses in age-related macular degeneration. *Invest. Ophthalmol. Vis. Sci.* 56 3976–3983. 26087362 10.1167/iovs.15-17013

[B32] Lynch-Day M. A. Mao K. Wang K. Zhao M. Klionsky D. J. (2012). The role of autophagy in Parkinson’s disease. *Cold Spring Harb. Perspect. Med.* 2:a009357. 10.1101/cshperspect.a009357 PMC3312403 22474616

[B33] Maguire M. G. Martin D. F. Ying G.-S. Jaffe G. J. Daniel E. Grunwald J. E. (2016). Five-Year outcomes with anti–vascular endothelial growth factor treatment of neovascular age-related macular degeneration: The comparison of age-related macular degeneration treatments trials. *Ophthalmology* 123 1751–1761. 27156698 10.1016/j.ophtha.2016.03.045 PMC4958614

[B34] Miller R. L. James-Kracke M. Sun G. Y. Sun A. Y. (2009). Oxidative and Inflammatory Pathways in Parkinson’s Disease. *Neurochem. Res.* 34 55–65. 18363100 10.1007/s11064-008-9656-2

[B35] Mitchell P. Liew G. Gopinath B. Wong T. Y. (2018). Age-related macular degeneration. *Lancet* 392 1147–1159. 30303083 10.1016/S0140-6736(18)31550-2

[B36] Mohana Devi S. Mahalaxmi I. Aswathy N. P. Dhivya V. Balachandar V. (2020). Does retina play a role in Parkinson’s Disease? *Acta Neurol Belg.* 120 257–265. 31965540 10.1007/s13760-020-01274-w

[B37] Moschos M. M. Tagaris G. Markopoulos I. Margetis I. Tsapakis S. Kanakis M. (2011). Morphologic changes and functional retinal impairment in patients with Parkinson disease without visual loss. *Eur. J. Ophthalmol.* 21 24–29. 10.5301/ejo.2010.1318 20602324

[B38] Nowacka B. Lubinski W. Honczarenko K. Potemkowski A. Safranow K. (2014). Ophthalmological features of Parkinson disease. *Med. Sci. Monit.* 20 2243–2249. 25387009 10.12659/MSM.890861 PMC4238794

[B39] Nowacka B. Lubinski W. Honczarenko K. Potemkowski A. Safranow K. (2017). Glaucoma in Patients with Parkinson’s Disease. *J. Alzheimers Dis. Parkinsonism* 7 301–303.

[B40] Ortuño-Lizarán I. Beach T. G. Serrano G. E. Walker D. G. Adler C. H. Cuenca N. (2018). Phosphorylated α-synuclein in the retina is a biomarker of Parkinson’s disease pathology severity. *Mov. Disord.* 33 1315–1324. 10.1002/mds.27392 29737566 PMC6146055

[B41] Ortuño-Lizarán I. Sãnchez-Sãez X. Lax P. Serrano G. E. Beach T. G. Adler C. H. (2020). Dopaminergic retinal cell loss and visual dysfunction in Parkinson Disease. *Ann. Neurol.* 88 893–906. 32881029 10.1002/ana.25897 PMC10005860

[B42] Pajares M. Rojo A. I. Manda G. Boscã L. Cuadrado A. (2020). Inflammation in Parkinson’s disease: Mechanisms and therapeutic implications. *Cells* 9:1687. 10.3390/cells9071687 PMC7408280 32674367

[B43] Park S. J. Kwon K. E. Choi N. K. Park K. H. Woo S. J. (2015). Prevalence and incidence of exudative age-related macular degeneration in South Korea: A nationwide population-based study. *Ophthalmology* 122 2063–2070.e1. 26208437 10.1016/j.ophtha.2015.06.018

[B44] Pennington K. L. DeAngelis M. M. (2016). Epidemiology of age-related macular degeneration (Amd): Associations with cardiovascular disease phenotypes and lipid factors. *Eye Vis.* 3:34. 10.1186/s40662-016-0063-5 PMC5178091 28032115

[B45] Poewe W. Seppi K. Tanner C. M. Halliday G. M. Brundin P. Volkmann J. (2017). Parkinson disease. *Nat. Rev. Dis. Primers* 3:17013. 10.1038/nrdp.2017.13 28332488

[B46] Quan H. Sundararajan V. Halfon P. Fong A. Burnand B. Luthi J. C. (2005). Coding algorithms for defining comorbidities in Icd-9-Cm and Icd-10 administrative data. *Med. Care* 43 1130–1139. 16224307 10.1097/01.mlr.0000182534.19832.83

[B47] Rim T. H. Yoo T. K. Kim S. H. Kim D. W. Kim S. S. (2019). Incidence of exudative age-related macular degeneration and treatment load under the Korean national health insurance system in 2010-2015. *Br. J. Ophthalmol.* 103 1361–1366. 10.1136/bjophthalmol-2018-312693 30573498

[B48] Rudnicka A. R. Kapetanakis V. V. Jarrar Z. Wathern A. K. Wormald R. Fletcher A. E. (2015). Incidence of late-stage age-related macular degeneration in American whites: Systematic review and meta-analysis. *Am. J. Ophthalmol.* 160 85–93.e3. 10.1016/j.ajo.2015.04.003 25857680

[B49] Salimiaghdam N. Riazi-Esfahani M. Fukuhara P. Schneider K. Kenney C. (2019). Age-related Macular Degeneration (Amd): A review on its epidemiology and risk factors. *Open Ophthalmol. J.* 13 90–99.

[B50] Santos-García D. de la Fuente-Fernãndez R. (2013). Impact of non-motor symptoms on health-related and perceived quality of life in Parkinson’s disease. *J. Neurol. Sci.* 332 136–140. 23890935 10.1016/j.jns.2013.07.005

[B51] Satue M. Rodrigo M. J. Obis J. Vilades E. Gracia H. Otin S. (2017). Evaluation of progressive visual dysfunction and retinal degeneration in patients With Parkinson’s Disease. *Invest. Ophthalmol. Vis. Sci.* 58 1151–1157. 10.1167/iovs.16-20460 28208185

[B52] Satue M. Seral M. Otin S. Alarcia R. Herrero R. Bambo M. P. (2014). Retinal thinning and correlation with functional disability in patients with Parkinson’s disease. *Br. J. Ophthalmol.* 98 350–355. 24276697 10.1136/bjophthalmol-2013-304152

[B53] Srinivasan S. Swaminathan G. Kulothungan V. Ganesan S. Sharma T. Raman R. (2017). Age-related macular degeneration in a South Indian population, with and without diabetes. *Eye* 31 1176–1183. 10.1038/eye.2017.47 28387762 PMC5558219

[B54] Thomas C. J. Mirza R. G. Gill M. K. (2021). Age-related macular degeneration. *Med. Clin. North Am.* 105 473–491. 33926642 10.1016/j.mcna.2021.01.003

[B55] Tugcu B. Melikov A. Yildiz G. B. Gøkcal E. Ercan R. Uysal O. (2020). Evaluation of retinal alterations in Parkinson disease and tremor diseases. *Acta Neurol. Belg.* 120 107–113. 31679150 10.1007/s13760-019-01228-x

[B56] Voutilainen-Kaunisto R. M. Teræsvirta M. E. Uusitupa M. I. Niskanen L. K. (2000). Age-related macular degeneration in newly diagnosed type 2 diabetic patients and control subjects: A 10-year follow-up on evolution, risk factors, and prognostic significance. *Diabetes Care* 23 1672–1678. 10.2337/diacare.23.11.1672 11092291

[B57] Wong W. L. Su X. Li X. Cheung C. M. Klein R. Cheng C. Y. (2014). Global prevalence of age-related macular degeneration and disease burden projection for 2020 and 2040: A systematic review and meta-analysis. *Lancet Glob. Health* 2 e106–e116. 10.1016/S2214-109X(13)70145-1 25104651

[B58] Yongpeng Z. Yaxing W. Jinqiong Z. Qian W. Yanni Y. Xuan Y. (2022). The association between diabetic retinopathy and the prevalence of age-related macular degeneration-the Kailuan Eye Study. *Front. Public Health* 10:922289. 10.3389/fpubh.2022.922289 35923972 PMC9339787

[B59] Young J. B. Godara P. Williams V. Summerfelt P. Connor T. B. Tarima S. (2019). Assessing retinal structure in patients with Parkinson’s Disease. *J. Neurol. Neurophysiol.* 10:485. 10.4172/2155-9562.1000485 PMC6494090 31057987

[B60] Yuk J.-S. Hwang J. H. (2022). Menopause and the risk of developing age-related macular degeneration in Korean women. *J. Clin. Med.* 11:1899. 10.3390/jcm11071899 35407510 PMC8999594

